# Pharmacologic Induction of Endotoxin Tolerance in Dendritic Cells by L-Kynurenine

**DOI:** 10.3389/fimmu.2020.00292

**Published:** 2020-03-11

**Authors:** Giorgia Manni, Giada Mondanelli, Giulia Scalisi, Maria Teresa Pallotta, Dario Nardi, Eleonora Padiglioni, Rita Romani, Vincenzo Nicola Talesa, Paolo Puccetti, Francesca Fallarino, Marco Gargaro

**Affiliations:** Department of Experimental Medicine, University of Perugia, Perugia, Italy

**Keywords:** L-kynurenine, dendritic cells, endotoxin tolerance, aryl hydrocarbon receptor, indoleamine (2, 3)-dioxygenase

## Abstract

Endotoxin tolerance aims at opposing hyperinflammatory responses to lipopolysaccharide (LPS) exposure. The aryl hydrocarbon receptor (AhR) participates in protection against LPS-mediated tissue damage, as it plays a necessary role in restraining the proinflammatory action of IL-1β and TNF-α while fostering the expression of protective TGF-β. TGF-β, in turn, promotes durable expression of the immune regulatory enzyme indoleamine 2,3-dioxygenase 1 (IDO1). IDO1 degrades L-tryptophan to L-kynurenine—an activating ligand for AhR—thus establishing a feed-forward loop. In this study, we further demonstrate that L-kynurenine also promotes the dissociation of the Src kinase–AhR cytosolic complex, leading to the activation of both genomic and non-genomic events in conventional dendritic cells (cDCs) primed with LPS. Specifically, the Src kinase, by phosphorylating the downstream target IDO1, triggers IDO1’s signaling ability, which results in enhanced production of TGF-β, an event key to establishing full endotoxin tolerance. We demonstrated that exogenous L-kynurenine can substitute for the effects of continued or repeated LPS exposure and that the AhR–Src–IDO1 axis represents a critical step for the transition from endotoxin susceptibility to tolerance. Moreover, much like fully endotoxin-tolerant dendritic cells (DCs) (i.e., treated twice with LPS *in vitro*), DCs—treated once with LPS *in vitro* and then with kynurenine—confer resistance on naïve recipients to an otherwise lethal LPS challenge. This may have clinical implications under conditions in which pharmacologically induced onset of endotoxin tolerance is a therapeutically desirable event.

## Introduction

A first exposure to lipopolysaccharide (LPS) makes mice resistant to shock caused by a subsequent LPS injection, an occurrence known as LPS or endotoxin tolerance. In particular, a low amount of LPS causes a long-lived state of cell refractoriness to a second LPS challenge that prevents excessive inflammatory responses. Appropriate modulation of LPS-responsive genes to foster the onset of endotoxin tolerance would be beneficial in clinical settings dominated by acute hyperinflammatory responses to infection (1, 2).

In infection resistance and disease tolerance to microbial insult, the ligand-activated transcription factor aryl hydrocarbon receptor (AhR) adaptively balances aggressive immune responses (“resistance”) with the host’s ability to withstand the negative effects of infection, namely, immunopathology or damage due to pathogen metabolism and virulence factors (“tolerance”) (3, 4).

AhR is a heterodimeric transcriptional regulator. Broadly present in a variety of animal species as well as in humans, AhR has constitutive functions that are only now being appreciated in their intricacies. The main interest is now shifting from the role of AhR in the hepatic metabolism—and thus inactivation—of potentially toxic xenobiotics toward the nature of its physiological ligands as well as its mode of action in response to functionally distinct molecules, which are remarkably different in nature as to their endogenous source and chemical structure. Among physiologically relevant ligands, tryptophan metabolites, including L-kynurenine, act as activating molecules for the receptor (3, 5).

Animal and human data indicate that AhR is key to multiple signaling pathways critical to cellular and tissue homeostasis, which relates apparently disparate aspects of physiology, including cell proliferation and differentiation, gene regulation, cell motility and migration, inflammation, and neoplasia (6–9).

In its inactive state, AhR is complexed with two heat-shock proteins 90 (Hsp90), p23, the AhR-interacting protein (AIP), and the non-receptor tyrosine kinase c-Src (10). Upon ligand binding, the chaperones are released, and AhR shuttles to the nucleus, where it heterodimerizes with the AhR nuclear translocator (ARNT) at promoter recognition sequences of target genes.

Beside these genomic events, AhR mediates non-canonical signaling pathways. AhR can indeed participate in the phosphorylation of multiple target proteins through the release of c-Src from the cytoplasmic complex (11), and it can also regulate protein turnover thanks to its E3 ubiquitin ligase activity (12). The immunoregulatory enzyme indoleamine 2,3-dioxygenase (IDO1) has been identified as one of the c-Src targets (13). In particular, double exposure of splenic dendritic cells (DCs) to LPS triggers AhR–Src kinase-dependent IDO1 phosphorylation and TGF-β production, and IDO1 expression by DCs further increases L-kynurenine production, the main tryptophan byproduct (3). Based on these data, we investigated whether exogenous L-kynurenine might be able to replace the second LPS exposure for the transition from endotoxin susceptibility to tolerance. Here we found that L-kynurenine can replace a second exposure to LPS in triggering genomic and non-genomic, AhR-dependent effects capable of rendering conventional DCs (cDCs) suitable for transferring endotoxin tolerance onto naïve recipients. In clinical settings where onset of endotoxin tolerance is therapeutically desirable, replacing the LPS stimulus by a pharmacological means such as L-kynurenine would represent an undoubtful advantage in terms of patients’ safety and possible side effects.

## Materials and Methods

### Mice

Eight- to 10-week-old male C57BL/6 mice were obtained from Charles River Breeding Laboratories. IDO1-deficient mice (*Ido1*^–^*^/^*^–^) were purchased from The Jackson Laboratory. B6.129-Ahrtm1Bra/J Ahr-deficient (*Ahr*^–^*^/^*^–^) mice were kindly supplied by B. Stockinger (MRC National Institute for Medical Research, London, United Kingdom). All *in vivo* studies were in compliance with national (Italian Parliament DL 116/92) and Perugia University Animal Care and Use Committee guidelines, and the overall study was approved by the Bioethics Committee of the University of Perugia.

### Cell Lines and Cell Culture

The mouse fibroblast line, SYF, was obtained from ATCC, and cells were cultured in DMEM (Gibco) supplemented with 10% FBS at 37°C. This cell line was transfected to over-express c-Src and AhR and used for immunoprecipitation assays and as a source of nuclear extracts, as described below. Mouse embryonic fibroblasts (MEFs) were obtained according to the guidelines of the University of Perugia Ethical Committee and the European Communities Council Directive 2010/63/EU. MEFs and *Ahr*^–^*^/^*^–^ MEFs were prepared as described (14). Briefly, pregnant mice were sacrificed at 13.5 days post-coitum (dpc) by cervical dislocation. The embryos were separated from placenta and membranes and were placed in 10 cm culture dishes in sterile phosphate-buffered saline. Then, the liver, heart, and brain were removed and discarded. The remaining part of each embryo was washed and minced with cool razor blades and incubated 20 min at 37°C with trypsin-EDTA (500 mg L^–1^). The minced tissues were chopped by repeated pipetting, and then the cell suspension was plated on 10 cm tissue culture dishes, and DMEM medium (10 ml) containing 10% FBS (Euroclone) was added. Electroporation was used to transfect MEFs.

### DC Preparation

Dendritic cells were isolated from bone marrows of C57BL/6 mice by crushing tibias, femurs, and hips in MACS buffer as previously described (15). Extracted cells were filtered through a 40 μm cell strainer and centrifuged at 300 × *g* for 5 min. Cells were re-suspended in red blood cell lysis buffer to remove erythrocytes. For DC differentiation, BM cells were cultured at a density of 2 × 10^6^/ml in culture media (IMDM from Gibco) conditioned with 5% Flt-3l for 9 days at 37°C with 7% CO_2_. At the end of the culture, 25–30 million/mouse total DCs were obtained. Purification of cDCs was performed by MACS column (Miltenyi) and biotin antibodies (Biolegend). Total DCs were incubated with biotin mouse monoclonal antibodies against B220 (a marker of pDCs and B cells). After this, cells were incubated with MagniSort Streptavidin Negative Selection Beads (Thermo Fisher) followed by depletion of B220^+^ cells. We collected the B220^–^ cell fraction and used this to verify cell purity. cDCs were stained with fluorescent antibodies, identified as B220^–^ CD3^–^ and MHC-II^+^ CD11c^+^ CD24^+^ CD172^+^ by cytofluorimetric analysis by LSRFortessa (BD BioSciences), and analyzed by flowJo data analysis software. Cell purity was more than 90%. Cells (1 × 10^6^/ml) were primed with 250 ng/ml LPS (055:B5 Sigma-Aldrich) overnight before treating with II° LPS (1 μg/ml) or 50 μM L-kynurenine (Sigma-Aldrich) for 24 h.

### Cytokine Production

Purified DCs were re-suspended at 1 × 10^6^/ml in fresh media in the presence or absence of LPS and L-kynurenine for a total of 36 h; supernatants were collected and analyzed for TGF-β by ELISA according to the manufacturer’s instructions (R&D system). DCs treated with LPS with either LPS or L-kynurenine were analyzed by ELISA for IL-10 and TGF-β contents according to the manufacturer’s instructions (R&D system) (16).

### Real-Time PCR

Real-time PCR (for mouse *Ido1*, *Ido2*, and *Tdo2* expressions) analyses were carried out as described (17) using the specific primers listed in Supplementary Table S1. In all figures depicting RT-PCR data, bars represent the ratio of the relevant gene to β-actin-encoding gene expression, as determined by the relative quantification method (ΔΔCT; means ± SD of triplicate determinations).

### Co-immunoprecipitation

SYF cells expressing c-Src kinase and LPS-primed cDCs were stimulated with L-kynurenine at 50 μM for different lengths of time (0.5, 1, and 2 h). Nuclear and cytoplasmic fractions were prepared from cells lysed on ice with Buffer N (15 mM Tris–HCl, pH 7.5, 15 mM NaCl, 60 mM KCl, 5 mM MgCl_2_, 25 mM sucrose, 0.6% Non-idet P-40, 1 mM DTT, 2 mM Na_3_VO_4_). Lysates were then immunoprecipitated by means of sheep polyclonal antibody recognizing AhR, previously complexed with G Dynabeads (Invitrogen). Alternatively, cytosolic and nuclear lysates were run directly on SDS/PAGE.

### Western Blotting

IDO1 and pIDO1 expressions were investigated in cDCs by immunoblot with a rabbit monoclonal anti-mouse IDO1 antibody (cv152) or a rabbit polyclonal antibody to the phosphorylated immunoreceptor tyrosine-based inhibitory motifs (ITIM) motif of IDO1, respectively, both raised in our laboratory (18). c-Src and its phosphorylated form were revealed by specific anti-Src and -pSrc antibodies (Tyr416; Cell Signaling Technology). Anti-AhR antibody was from R&D system. Anti-β-actin and β-tubulin antibodies (Sigma-Aldrich) were used as a normalizer. Anti-lamininB antibody (Thermo Fisher) was used as a nuclear extract control. Whole-cell extracts, immunoprecipitates, and nuclear and cytosolic extracts were denatured in Laemmli sample buffer at 95°C for 5 min. Samples were run on a gel and transferred onto a nitrocellulose membrane (Bio-Rad). Immunoblots were blocked in TBS containing 3% BSA or 5% non-fat milk and 0.1% Tween 20 at room temperature for 1 h and then incubated with primary antibody overnight at 4°C. After washing, membranes were incubated with goat anti-rabbit, goat anti-mouse IgG or anti-sheep IgG conjugated to horseradish peroxidase (Jackson ImmunoResearch Laboratories, Inc) for 1 h at room temperature in 5% non-fat milk and 0.1% Tween 20 TBS. Then, membranes were washed and developed with ECL BioRad.

### cDC–T-Cell Co-culture

LPS-unprimed and LPS-primed cDCs treated or not with L-kynurenine, as described above, were cultured with CD4^+^ T cells isolated from the spleens of Foxp3^YFP^ mice. Briefly, CD4^+^ T cells were isolated via cell separation by two steps. A first step was meant to enrich the CD3^+^ cell fraction, using biotin mouse monoclonal antibodies against B220 (a marker of pDCs and B cells) and CD11c (a marker of DCs). After this step, cells were incubated with MagniSort Streptavidin Negative Selection Beads (Thermo Fisher) followed by depletion of B220^+^CD11c^+^. B220^–^ CD11c^–^ collected cell fractions were incubated with magnetic anti-mouse CD4 beads (Miltenyi Biotec) to select CD4^+^ T cells. The purity of CD4^+^ T cells was verified by FACS analysis by cell staining with anti-B220, anti-CD3, anti-CD11c, anti-CD4, and anti-CD8 specific antibodies. CD4^+^ T cells (2 × 10^5^) were activated with 5 μg/ml anti-CD3 mAb (clone OKT3) and co-cultured for 4 days with unprimed and primed cDCs (5 × 10^4^) treated or not with L-kynurenine for 36 h (19). Treg cells were evaluated by FACS, analyzing the induction of Foxp3 expressing YFP.

### Endotoxin Tolerance Mouse Model and Histology

In the induction of primary endotoxemia, mice of the different genotypes (WT, *Ahr*^–^*^/^*^–^, and *Ido1*^–^*^/^*^–^) were randomly grouped (10 per group) and injected intravenously with 1 × 10^6^ cDCs, primed or not with LPS and treated or not with L-kynurenine, as described above. After 48 h, mice were injected intraperitoneally with a lethal dose of LPS (40 mg/kg). Vehicle-treated groups (no cDCs) were used as controls. Animals were monitored daily for 1 week for mortality or the presence of signs of moribundity, including lack of responsiveness to manual stimulation, immobility, and inability to eat or drink.

For histology, morphological analysis of paraffin-embedded lung sections (4 μm) from mice treated as above included stained with H&E at 48 h after challenge and examination by light microscopy, using representative specimens from one of three experiments.

### Statistical Analysis

For animal studies, randomization consisted of generating a random permutation of a sequence and selecting random numbers. On assessing outcomes involving scores, the investigator was totally blinded to group allocation. The log-rank test was used for paired data analyses of Kaplan–Meier survival curves. All *in vitro* determinations are means ± SD from three independent experiments and were evaluated by two-way and one-way ANOVA. GraphPad Prism version 6.0 (San Diego, CA, United States) was used for all analyses and graph preparation.

## Results

### L-Kynurenine Potentiates IDO1 Induction and TGF-β Production in LPS-Primed cDCs via AhR

**FIGURE 1:**
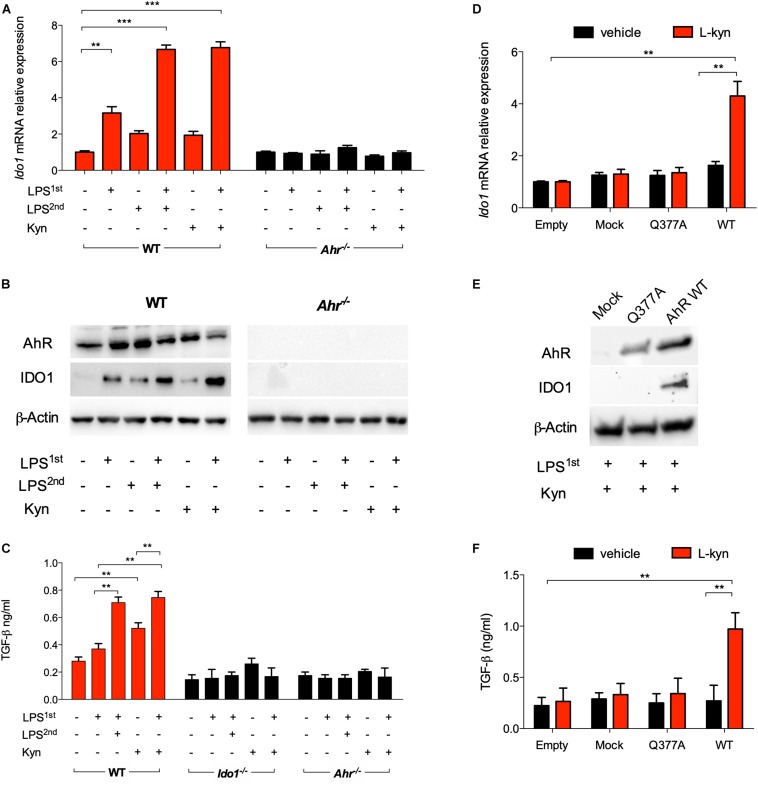
L-Kynurenine induces indoleamine 2,3-dioxygenase 1 (IDO1) and TGF-β in lipopolysaccharide (LPS)-primed conventional dendritic cells (cDCs) *via* aryl hydrocarbon receptor (AhR). **(A)** Real-time PCR analysis of *Ido1* mRNA expression in WT and *Ahr*^−/−^ BMDCs, either unprimed or primed with LPS (250 ng/ml) overnight (LPS1st) and then treated with a second dose of LPS (1 μg/ml) (LPS2nd) or L-kynurenine (50 mM) for an additional 24 h. Data (mean ± SD of three experiments) are represented as normalized transcript expression in the samples relative to normalized transcript expression in control cultures. **(B)** Western blotting analysis of IDO1 and AhR protein expression in cells treated as in A. β-Actin was used as a loading control. **(C)** TGF-β content was measured in 48 h culture supernatants from either WT, *Ido1*^−/−^, or *Ahr*^−/−^ cDCs stimulated as in A, after removing the stimuli by extensive washing, followed by additional culturing for 48 h in medium alone. **(D)** Real-time PCR analysis of *Ido1* mRNA expression in AhR-deficient cDCs reconstituted with mutated AhR (bearing the Q377A mutation) or the empty vector (mock). Cells were primed with LPS, followed by L-kynurenine treatment or vehicle alone. Data (mean ± SD of three experiments) are represented as normalized transcript expression in the samples relative to normalized transcript expression in control cultures. **(E)** Western blotting analysis of IDO1 and AhR protein expression in cells treated as in **(D)**. β-Actin was used as a loading control. **(F)** TGF-β production was measured in cells treated as in **(D)**. **P < 0.01, ***P < 0.001 (two-way ANOVA).

Unlike a single LPS stimulation, repeated exposure *in vitro* of splenic cDCs to endotoxin significantly induces IDO1 protein expression and L-kynurenine production, the main IDO1 enzymatic product. Such conditioned cDCs protect mice from lethal endotoxemia, and this effect requires that the cDCs be competent for IDO1 and TGF-β production (17). We here examined whether L-kynurenine could substitute for the second LPS challenge in mimicking an endotoxin-tolerant state in cDCs. We preliminarily set up cultures of bone marrow-derived cDCs (Supplementary Figure S1A). Then, IDO1 induction was assessed in these cells after priming with LPS overnight, followed by treatment with LPS or L-kynurenine for an additional 24 h, and unprimed cDCs were used as control. Priming with LPS alone modestly induced IDO1 at both transcript and protein levels; however, sequential treatment with LPS and L-kynurenine significantly enhanced this effect. In LPS-primed cDCs derived from AhR-deficient mice, IDO1 induction by L-kynurenine as a second stimulus was instead lost (Figures 1A,B). Under the same conditions, the expressions of the IDO1 paralog, *Ido2*, and of *Tdo*2 were not significantly affected by the treatment with LPS and L-kynurenine. Of note, *Ido2* expression was induced by LPS alone on first exposure but not by a subsequent exposure to L-kynurenine or LPS as a repeated stimulus (Supplementary Figure S1B). Treatment of cDCs with L-kynurenine in the absence of prior LPS did not affect *Ido1* expression (Figure 1A), implying that early TLR4-dependent events are necessary for initiating the loop whereby L-kynurenine will later reinforce AhR-dependent *Ido1* transcription. The transition from endotoxin susceptibility to tolerance in cDCs is marked by an increased TGF-b secretion (3). We found that in unprimed cDCs, L-kynurenine alone treatment would increase TGF-β production according to a slower kinetic pattern than when used after LPS pre-conditioning. The ideal conditions for TGF-β production for the former involved removing L-kynurenine by extensive washing, followed by additional culturing for 48 h in medium alone (Figure 1C). Yet, production of the cytokine was strongly enhanced by previous LPS conditioning (Figure 1C). As expected, TGF-β production was unaffected by LPS + L-kynurenine treatment in cDCs derived from *Ahr*^–^*^/^*^–^ and *Ido1*^–^*^/^*^–^ mice.

Based on the finding that L-kynurenine binds AhR on the Gln377 residue, and in order to validate the ability of L-kynurenine to induce IDO1 *via* AhR in cDCs, we reconstituted AhR-deficient cDCs with a vector encoding the mutated form of AhR where Gln at position 377 is replaced by an Ala residue (Q377A) as described (3). The engineered cDCs were primed with LPS and then treated with L-kynurenine. Cells, either reconstituted with empty vector or the Q377A mutant, failed to express IDO1 (Figures 1D,E) and to produce TGF-β (Figure 1F). While confirming the binding mode of L-kynurenine to the Gln377 domain of the PAS-B domain of AhR, these results showed a crucial requirement for the Gln377 residue of AhR in L-kynurenine-mediated induction of IDO1 in LPS-primed cDCs, and for reprogramming those cells toward a tolerogenic phenotype. Overall, these data suggest that, *in vitro*, externally added L-kynurenine contributes to reprogramming gene expression—including *Ido1*’s—in endotoxin tolerance via effects involving AhR.

### L-Kynurenine Induces AhR/Src-Dependent Phosphorylation of IDO1

**FIGURE 2:**
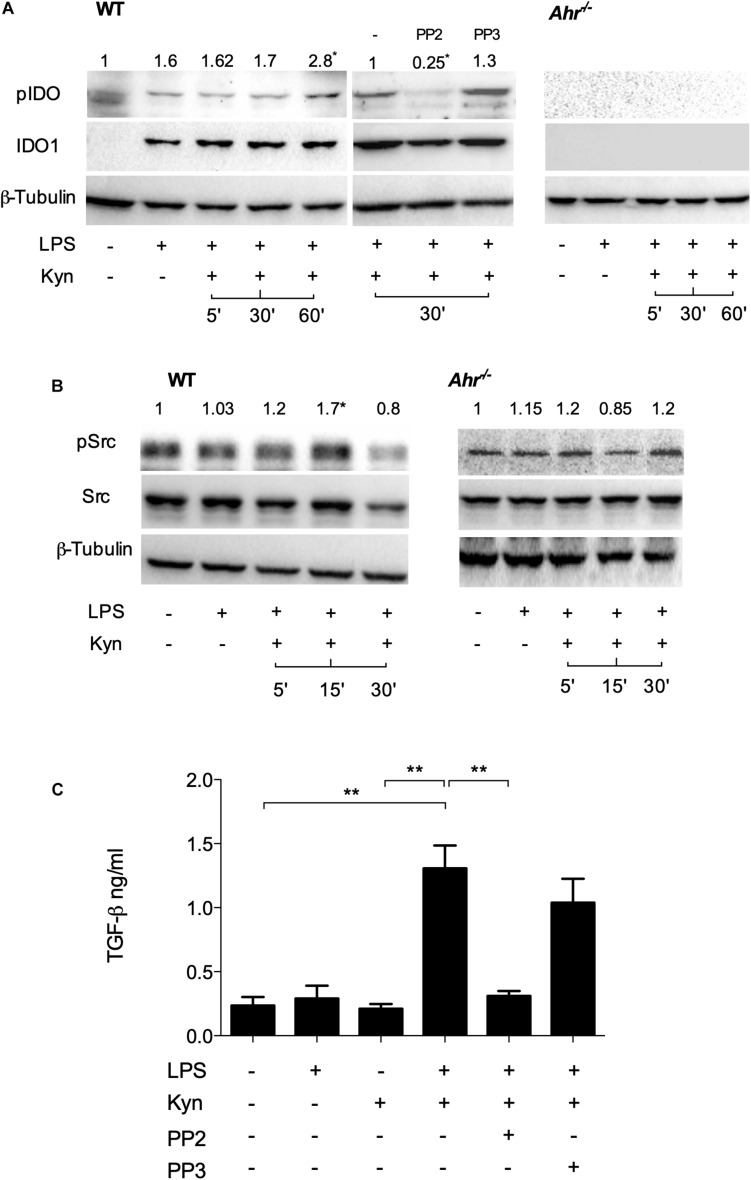
L-Kynurenine induces IDO1 phosphorylation in AhR- and Src-dependent manner. **(A)** Western blotting analysis of IDO1 phosphorylation in WT and AhR-deficient cDCs, either unprimed or primed with LPS and then treated with L-kynurenine alone or with L-kynurenine plus PP2 or PP3 (i.e., a c-Src kinase inhibitor and its inactive analog, respectively). One experiment of three is shown. pIDO/tubulin ratios were obtained by densitometric quantification of the relevant bands and are expressed relative to untreated cells **(B)**. Immunoblot analysis of Src and phosphorylated Src-Y416 (pSrc) in WT and AhR-deficient cDCs treated as in **(A)**. One experiment of three is shown. pSrc/tubulin ratios were obtained by densitometric quantification of the relevant bands and are expressed relative to untreated cells **(C)**. Production of TGF-β by cDCs treated as in **(A)**. Means ± SD from three experiments. **P < 0.01 (two-way ANOVA).

Phosphorylation of a specific domain in IDO1 is involved in its non-enzymatic function leading to reprogramming gene expression and the induction of a stable regulatory phenotype in splenic plasmacytoid DCs that will produce less IL-6 and more TGF-β in response to TLR signaling (20). We investigated whether IDO1 phosphorylation occurs in LPS-primed, IDO1-competent cDCs upon L-kynurenine exposure. We found that phosphorylation of IDO1 occurs after 30 min of L-kynurenine treatment in wild type cDCs but not in AhR-deficient cells (Figure 2A). The inhibition of c-Src—the most widely represented kinase in mouse cDCs (13)—with PP2 (but not its inactive analog PP3) negated IDO1 phosphorylation in LPS-primed cDCs exposed to L-kynurenine (Figure 2A).

It has been previously demonstrated that several AhR ligands can promote the activation of c-Src (21). We thus assessed c-Src activity by measuring the phosphorylation status of the kinase in LPS-primed cDCs upon challenge with L-kynurenine. Western blot analysis showed that c-Src activity increased at 5 and 15 min of L-kynurenine exposure (Figure 2B), an effect that was contingent on AhR activation in that it was lost in AhR-deficient cDCs. Moreover, we found that induction of c-Src activity by L-kynurenine is necessary for TGF-β production (Figure 2C). Overall, these data indicated that L-kynurenine can mimic the effects of a second LPS challenge in inducing an endotoxin-tolerant state. In particular, L-kynurenine promotes the activation of c-Src and the subsequent phosphorylation of IDO1 in AhR-competent cDCs.

### L-Kynurenine Favors the Dissociation of c-Src From the AhR Complex in cDCs

**FIGURE 3:**
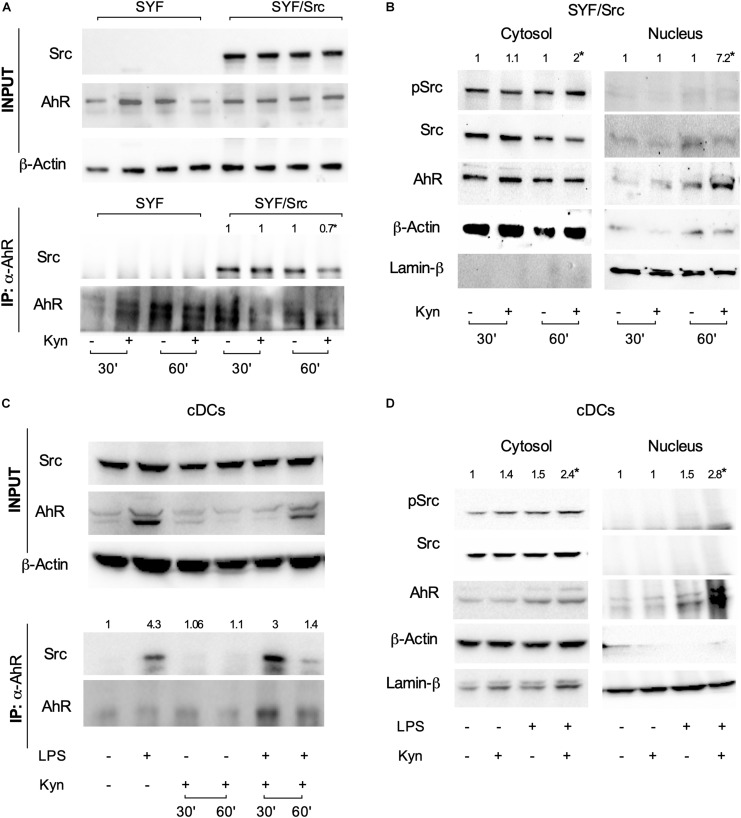
L-kynurenine promotes the activation of c-Src via AhR. **(A)** Immunoprecipitation of AhR from SYF cells, either reconstituted with c-Src or empty vector, and detection of Src and AhR by sequential immunoblotting with specific antibodies. Cells were treated with L-kynurenine for 30 and 60 min before lysis. Input refers to whole-cell lysates used as control of protein expression. One representative immunoblot of three is shown. **(B)** Expression of pSrc, Src, and AhR in nuclear and cytoplasmatic extracts from SYF cells expressing Src and treated as in **(A)**. Lamin-β and β-actin were used as loading control of nuclear and cytoplasmic extracts, respectively. One representative experiment of three is shown. **(C)** Immunoprecipitation of AhR from cDCs either unprimed or primed with LPS and then exposed to L-kynurenine for 30 and 60 min. Src and AhR protein expression were analyzed by sequential immunoblotting with specific antibodies. Input refers to whole-cell lysates used as control of protein expression. One representative experiment of three is shown. **(D)** Expression of pSrc, Src, and AhR in nuclear and cytoplasmatic extracts from cDCs treated as in **(C)** for 60 min. Lamin-β and β-actin were used as loading control of nuclear and cytoplasmic extracts, respectively. One representative experiment of three is shown. Src/AhR ratio **(A,C)** is calculated by densitometric quantification of the specific bands and is reported as fold change against untreated cells. pSrc/Src ratio [**(B,D)**; left panel, cytosol] and Ahr/Lamin-β ratio [**(B,D)**; right panel, nucleus] are measured by densitometric quantification of the specific bands and are expressed relative to untreated cells. Ratios are means from the three experiments. *P < 0.05 (one-way ANOVA).

The c-Src protein may exist in at least two different conformations (namely, inactive and active), whose relative stability determines the overall activity of the enzyme (22). Post-translational modifications, as well as binding of regulatory substrates and ligands, may alter this equilibrium by favoring one conformational population over the other and thus change the overall kinase activity. Several lines of evidence have shown that, upon tetrachlorodibenzo-*p*-dioxin (TCDD) binding, the kinase is released from the AhR complex and becomes activated (21). We thus investigated whether c-Src kinase activation mediated by L-kynurenine would require the dissociation from the AhR complex. SYF cells (i.e., a fibroblast cell line that does not express the ubiquitous c-Src, c-Fyn, and c-Yes kinases) were reconstituted with a vector coding for c-Src and exposed to L-kynurenine for different lengths of time. By co-immunoprecipitation assays, we found that AhR interacts with c-Src kinase and that L-kynurenine significantly reduces this association (Figure 3A). Moreover, by analyzing the cellular fractions of cells exposed to L-kynurenine, we observed that AhR localizes to the nucleus, while Src is mostly present in the cytosol in its active state (Figure 3B).

Co-immunoprecipitation and cellular fractionation studies confirmed that these molecular events (i.e., Src disjunction from the complex and AhR shuttling to the nucleus) occur in LPS-primed cDCs exposed to L-kynurenine as well (Figures 3C,D). Overall, these data demonstrated that L-kynurenine can indirectly regulate c-Src kinase activity. By binding AhR, L-kynurenine promotes the receptor’s conformational changes required for the release of c-Src, which then becomes activated and capable of phosphorylating downstream target proteins.

### L-Kynurenine-Primed cDCs Promote *in vitro* Induction of LAP^+^ T Cells

IDO1^+^ cDCs are known to be involved in the regulation of hyperinflammatory conditions *via* the generation of regulatory T cells (23). In particular, cDC co-treatment with LPS and L-kynurenine promotes TGF-β release in an AhR- and IDO1-dependent manner (Figure 2C) that is essential for Treg cell differentiation *in vitro* and *in vivo*.

**FIGURE 4:**
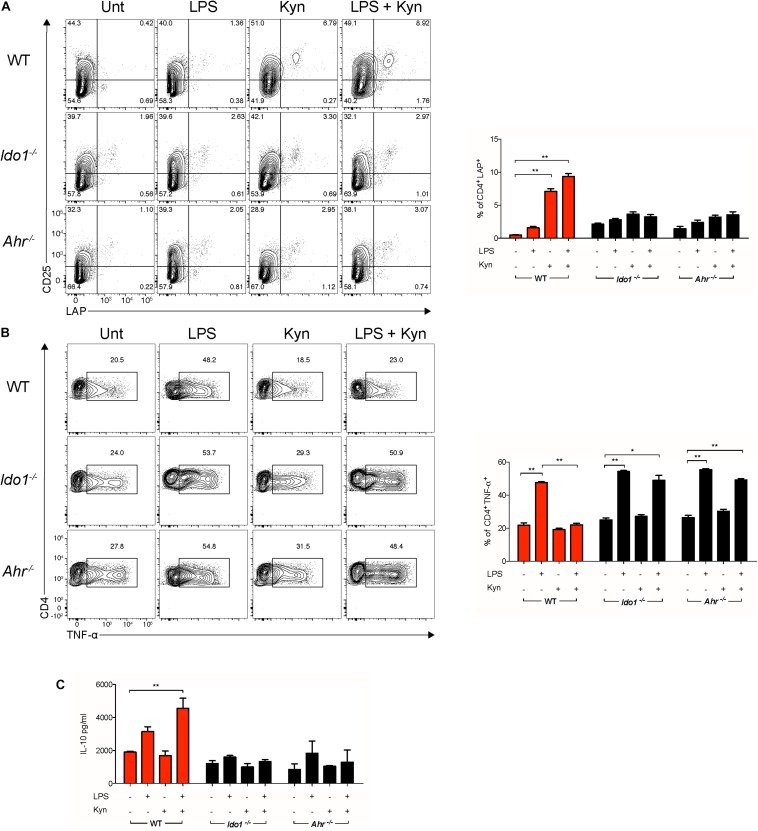
L-Kynurenine-primed cDCs promote *in vitro* induction of LAP^+^ T cells. Representative cytofluorimetric dot plots and relative analysis of percent expression of LAP **(A)** and TNF-α **(B)** coexpression in CD4^+^ cells co-cultured for 96 h with cDCs either unprimed or LPS-primed and then incubated for 24 h with Kyn at 50 μM. A mouse IgG2a antibody was used as isotype control. Shown in upper right quadrants are percentages of double-positive cells. **(C)** IL-10 production was measured in cells treated as in **(A)**. Data are means ± SD of three independent experiments. *P < 0.05, **P < 0.01 (two-way ANOVA).

To evaluate if the combined effects of L-kynurenine and LPS promoted functional regulatory cDCs, those cells—either unprimed or primed with LP—were incubated with L-kynurenine and cultured with naïve CD4^+^CD25^–^ T cells, after removing the stimuli by extensive washing. L-Kynurenine, either alone or in combination with LPS, significantly increased LAP expression on the T-cell surface relative to control samples (Figures 4A,B). By contrast, when the cDCs had been derived from AhR- and IDO1-deficient mice, this effect was abolished (Figures 4A,B). Foxp3 expression was evaluated in the same co-cultures. We failed to observe any differences between samples (data not shown). Moreover, the primed cDCs further treated with L-kynurenine decreased TNFα production as well as increased IL-10 secretion by the T cells in AhR- and IDO1-dependent fashion (Figure 4C). Overall, these findings validate the hypothesis that L-kynurenine progressively suppresses T CD4^+^ cell effector function, favoring instead the emergence of a regulatory T-cell phenotype.

### Adoptive Transfer of LPS-Primed cDCs Conditioned With L-Kynurenine *in vitro* Protects Mice From Lethal Endotoxemia

**FIGURE 5:**
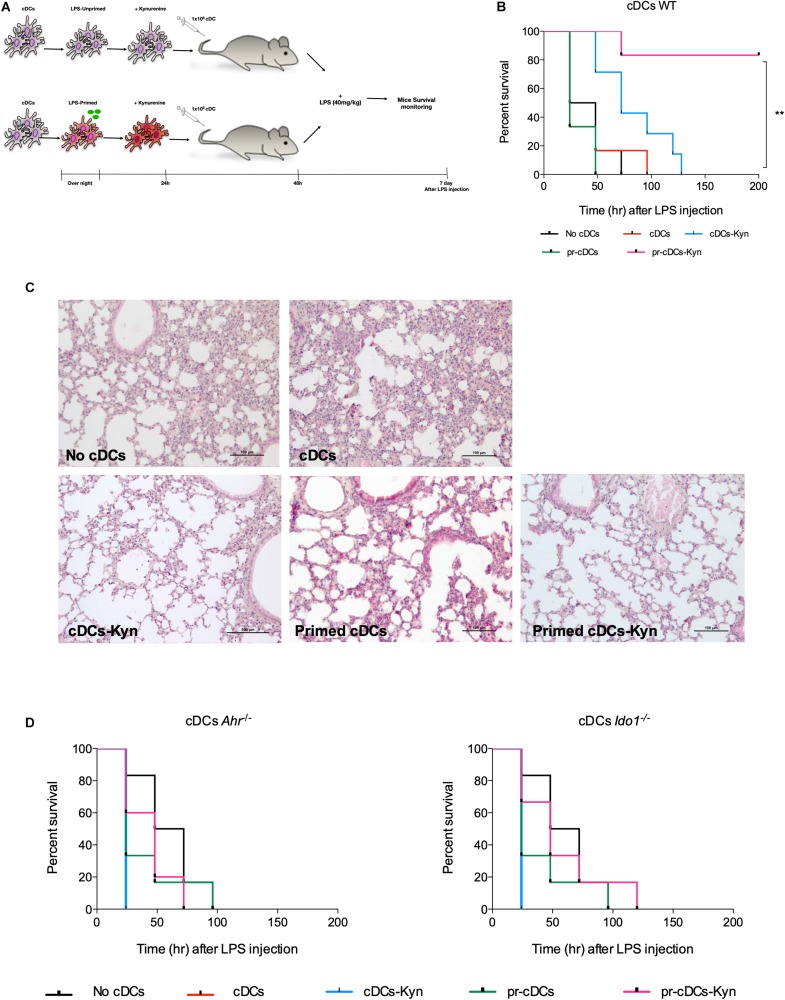
LPS-primed cDCs conditioned with L-kynurenine protect mice from lethal endotoxemia in AhR and IDO1-dependent manner. **(A)** Representative scheme of adoptive transfer mouse model. **(B)** Survival curves of WT mice receiving an otherwise lethal 40 mg/kg LPS challenge after transfer of cDCs. Mice were transferred with AhR-competent cDCs (1.5 × 10^6^/mouse), either unprimed (cDCs) or primed *in vitro* with LPS (1 μg/ml) and then stimulated with 50 μM kyn (pr-cDC–kyn) or medium (pr-cDCs) for an additional 24 h. Survival was monitored every 12 h through day 8 of LPS challenge. **(C)** Histopathology of lung obtained from mice treated as in **(B)**. Scale bar, 100 μm. **(D)** Survival curves of *Ahr*^−/−^ and *Ido1*^−/−^ mice receiving an otherwise lethal 40 mg/kg LPS challenge after transfer of cDCs *in vitro* treated as in **(A)**. *n* = 8–10 per group per experiment. One experiment representative of three. **P < 0.01 (log-rank test).

Because cDCs stimulated twice *in vitro* with LPS protect mice from a primary, otherwise lethal LPS challenge (3), we investigated whether the combined effects of LPS and L-kynurenine treatment *in vitro* would confer a protective potential on those cells *in vivo*. We established an adoptive transfer model using Flt-3L-induced, BM-derived cDCs (Supplementary Figure S1A). LPS-primed or unprimed cDCs, treated or not with L-kynurenine, were transferred i.v., into naïve mice to be challenged at 48 h with a lethal i.p., dose of LPS (Figure 5A). We found that all vehicle-treated control animals died within 48–72 h after LPS challenge. Among mice treated with differentially treated DCs, we found that as many as 80% mice receiving LPS + L-kynurenine-treated DCs survived challenge over 200 h (Figure 5B). To further assess the effects of cDCs *in vivo*, groups of mice were sacrificed at 48 h of LPS challenge, and lung injury—a typical feature of septic shock and endotoxemia—was sought for on gross pathology. Interestingly, lungs of mice receiving primed cDCs further conditioned by L-kynurenine were characterized by reduced inflammatory cell infiltration and vascular congestion relative to control or to the other DC treated groups (Figure 5C). Based on *in vitro* evidence of AhR and IDO1 dependence for pharmacologic induction of endotoxin tolerance by LPS and L-kynurenine in cDCs, the same experiment was performed using LPS-primed or unprimed cDCs, treated or not with L-kynurenine from AhR- and IDO1-deficient mice. The protective effect of the double (LPS + L-kynurenine) pulsed cDCs on recipient mice was lost when the transferred cells were not competent for AhR and IDO1 (Figure 5D). Thus, successful transfer of endotoxin tolerance by fully tolerogenic DCs requires that AhR and IDO1 be fully functional.

## Discussion

In the present studies, we expand upon our previous observations that endotoxin tolerance *in vivo*—a condition clinically desirable to prevent the detrimental effect endotoxemia—may be transferred to naïve hosts by adoptive transfer of cDCs repeatedly exposed to LPS *in vitro* (3, 17). In particular, our previous studies had shown markedly differential (and even dichotomic) effects of single vs. repeated exposure of cDCs to LPS *in vitro*. Namely, a single exposure to LPS failed to upregulate IDO1—one of the mediators of anti-inflammatory functions of DCs—and it was instead associated with high IL-6, which promotes inflammation and IDO1 ubiquitin degradation. In contrast, cDCs stimulated twice with LPS expressed high levels of IDO1 and of TGF-β. Adoptive transfer of double LPS-treated cDCs greatly improved the outcome of an otherwise lethal LPS challenge *in vivo*, Such protective effect required that the *in vivo* transferred cDCs be fully competent for IDO1 and the host for TGF-β production.

Here we found that L-kynurenine can replace a second exposure to LPS in triggering genomic and non-genomic, AhR-dependent effects, making cDCs suitable for transferring endotoxin tolerance onto naïve recipients. The effect of externally added L-kynurenine to LPS-conditioned cDCs resulted in the promotion *Ido1* transcriptional expression and in IDO1 phosphorylation and TGF-β production. One major finding in this study was that L-kynurenine, by binding AhR, will promote the release/activation of c-Src kinase, thus triggering IDO1 signaling ability in cDCs.

Our initial experiments along this direction provided evidence that L-kynurenine potentiates IDO1 induction and TGF-β production in LPS-primed cDCs *via* AhR. AhR-deficient cDCs transfected with a vector encoding a mutated form of AhR incapable of binding L-kynurenine (3) failed to express IDO1 and to produce TGF-β when sequentially treated with LPS and L-kynurenine *in vitro*. Next, we demonstrated that one major effect of the latter was to induce AhR/Src-dependent phosphorylation of IDO1. IDO1, indeed, exerts both short-term and long-term effects in the regulation of immune homeostasis and in preventing both (hyper)acute and chronic inflammatory responses (4, 24). IDO1 contains ITIMs that, once phosphorylated, bind protein tyrosine phosphatases (SHP-1 and SHP-2) and thus activate an immunoregulatory signaling in DCs. This mechanism promotes sustained IDO1 expression, which is particularly important in restraining chronic inflammation and autoimmunity. This dual—shorter term and longer term—IDO1 ability to restrain inflammation is thus to be traced to two distinct functions of the IDO1 protein, one enzymatic in nature (i.e., tryptophan degradation to kynurenine) and the other independent of its enzymic function. As mentioned above, once phosphorylated in a specific ITIM (namely, ITIM1) domain, IDO1 becomes capable of signaling ability and long-term genomic effects restraining the production of proinflammatory mediators and the emergence of regulatory T cells (25, 26). Of particular interest in this study was the finding that L-kynurenine favors the dissociation of c-Src from the AhR complex in cDCs, allowing the former to phosphorylate the IDO1’s ITIM2 domain and promoting the generation of T cells with a regulatory phenotype. Finally, we found that—much like double LPS-pulsed DCs—adoptive transfer of LPS-primed cDCs conditioned with L-kynurenine *in vitro* protected mice from lethal endotoxemia.

Overall, the combined effects of LPS and L-kynurenine on cDCs initiates a feed-forward loop—triggered by L-kynurenine and sustained by the combined actions of IDO1, AhR, and TGF-β—which represents a key event in preventing mortality in endotoxin tolerance *in vivo*. Pharmacologically induced endotoxin tolerance, i.e., by means of kynurenine-based immunotherapy, may thus be considered a clinical option in the control of hyperinflammatory responses.

## Data Availability Statement

The datasets generated for this study are available on request to the corresponding author.

## Ethics Statement

All *in vivo* studies were in compliance with National (Italian Parliament DL 116/92) and Perugia University Animal Care and Use Committee guidelines, and the overall study was approved by the Bioethics Committee of the University of Perugia.

## Author Contributions

GMa and FF designed and supervised the study as a whole. MG and GMo performed the majority of experiments. MP, RR, and EP helped with some experiments. GS and DN helped with the generation of gene constructs and with the *in vivo* experiments. VT provided some tools. MG, FF, and PP wrote the manuscript.

## Conflict of Interest

The authors declare that the research was conducted in the absence of any commercial or financial relationships that could be construed as a potential conflict of interest.

## References

[1] Quintana FJ Bohannon JK Hernandez A Enkhbaatar P Adams WL Sherwood ER. The immunobiology of toll-like receptor 4 agonists: from endotoxin tolerance to immunoadjuvants. *Shock.* (2013) 40:451–62. 10.1097/SHK.0000000000000042 23989337 PMC3919163

[2] O’Carroll C Fagan A Shanahan F Carmody RJ. Identification of a unique hybrid macrophage-polarization state following recovery from lipopolysaccharide tolerance. *J Immunol.* (2014) 192:427–36. 10.4049/jimmunol.1301722 24337373

[3] Bessede A Gargaro M Pallotta MT Matino D Servillo G Brunacci C Aryl hydrocarbon receptor control of a disease tolerance defence pathway. *Nature.* (2014) 511:184–90. 10.1038/nature13323 24930766 PMC4098076

[4] Gargaro M Pirro M Romani R Zelante T Fallarino F. Aryl hydrocarbon receptor-dependent pathways in immune regulation. *Am J Transplant.* (2016) 16:2270–6. 10.1111/ajt.13716 26751261

[5] Zelante T Iannitti RG Cunha C De Luca A Giovannini G Pieraccini G Tryptophan catabolites from microbiota engage aryl hydrocarbon receptor and balance mucosal reactivity via interleukin-22. *Immunity.* (2013) 39:372–85. 10.1016/j.immuni.2013.08.003 23973224

[6] Quintana FJ Sherr DH. Aryl hydrocarbon receptor control of adaptive immunity. *Pharmacol Rev.* (2013) 65:1148–61. 10.1124/pr.113.007823 23908379 PMC3799235

[7] Stockinger B Di Meglio P Gialitakis M Duarte JH. The aryl hydrocarbon receptor: multitasking in the immune system. *Annu Rev Immunol.* (2014) 32:403–32. 10.1146/annurev-immunol-032713-120245 24655296

[8] Wu PY Yu IS Lin YC Chang YT Chen CC Lin KH Activation of aryl hydrocarbon receptor by kynurenine impairs progression and metastasis of neuroblastoma. *Cancer Res.* (2019) 79:5550–62. 10.1158/0008-5472.CAN-18-3272 31431462

[9] Gargaro M Pirro M Manni G De Luca A Zelante T Fallarino F. Aryl hydrocarbon receptor: an environmental sensor in control of allergy outcomes. In: Schmidt-Weber C editor. *Allergy Prevention and Exacerbation. Birkhäuser Advances in Infectious Diseases.* Cham: Springer (2017). p. 167–89.

[10] Gutiérrez-Vázquez C Quintana FJ. Regulation of the immune response by the aryl hydrocarbon receptor. *Immunity.* (2018) 48:19–33. 10.1016/j.immuni.2017.12.012 29343438 PMC5777317

[11] Ghotbaddini M Cisse K Carey A Powell JB. Simultaneous inhibition of aryl hydrocarbon receptor (AhR) and Src abolishes androgen receptor signaling. *PLoS One.* (2017) 12:e0179844. 10.1371/journal.pone.0179844 28671964 PMC5495210

[12] Ohtake F Baba A Takada I Okada M Iwasaki K Miki H Dioxin receptor is a ligand dependent E3 ubiquitin ligase. *Nature.* (2007) 446:562–6. 10.1038/nature05683 17392787

[13] Mondanelli G Bianchi R Pallotta MT Orabona C Albini E Iacono A A relay pathway between arginine and tryptophan metabolism confers immunosuppressive properties on dendritic cells. *Immunity.* (2017) 46:233–44. 10.1016/j.immuni 28214225 PMC5337620

[14] Dolciami D Gargaro M Cerra B Scalisi G Bagnoli L Servillo G Binding mode and structure-activity relationships of ITE as an aryl hydrocarbon receptor (AhR) agonist. *ChemMedChem.* (2018) 13:270–9. 10.1002/cmdc.201700669 29266750

[15] Durai V Bagadia P Briseño CG Theisen DJ Iwata A Davidson JT IV Altered compensatory cytokine signaling underlies the discrepancy between Flt3^–/–^ and Flt3l^–/–^ mice. *J Exp Med.* (2018) 215:1417–35. 10.1084/jem.20171784 29572360 PMC5940266

[16] Romani R Manni G Donati C Pirisinu I Bernacchioni C Gargaro M S1P promotes migration, differentiation and immune regulatory activity in amniotic-fluid-derived stem cells. *Eur J Pharmacol.* (2018) 833:173–82. 10.1016/j.ejphar.2018.06.005 29886240 PMC6086338

[17] Fallarino F Pallotta MT Matino D Gargaro M Orabona C Vacca C LPS-conditioned dendritic cells confer endotoxin tolerance contingent on tryptophan catabolism. *Immunobiology.* (2015) 220:315–21. 10.1016/j.imbio.2014.09.017 25278421

[18] Volpi C Mondanelli G Pallotta MT Vacca C Iacono A Gargaro M Allosteric modulation of metabotropic glutamate receptor 4 activates IDO1-dependent, immunoregulatory signaling in dendritic cells. *Neuropharmacology.* (2016) 102:59–71. 10.1016/j.neuropharm 26522434 PMC4720030

[19] Gargaro M Vacca C Massari S Scalisi G Manni G Mondanelli G Engagement of nuclear coactivator 7 by 3-hydroxyanthranilic acid enhances activation of aryl hydrocarbon receptor in immunoregulatory dendritic cells. *Front Immunol.* (2019) 10:1973. 10.3389/fimmu.2019.01973 31481962 PMC6710348

[20] Volpi C Fallarino F Bianchi R Orabona C De Luca A Vacca C A GpC-rich oligonucleotide acts on plasmacytoid dendritic cells to promote immune suppression. *J Immunol.* (2012) 189:2283–9. 10.4049/jimmunol.1200497 22844124

[21] Dong B Cheng W Li W Zheng J Wu D Matsumura F FRET analysis of protein tyrosine kinase c-Src activation mediated via aryl hydrocarbon receptor. *Biochim Biophys Acta.* (2011) 1810:427–31. 10.1016/j.bbagen.2010.11.007 21145940 PMC3049970

[22] Roskoski R Jr. Src protein-tyrosine kinase structure, mechanism, and small molecule inhibitors. *Pharmacol Res.* (2015) 94:9–25. 10.1016/j.phrs.2015.01.003 25662515

[23] Grohmann U Puccetti P. The coevolution of IDO1 and AhR in the emergence of regulatory T-cells in mammals. *Front Immunol.* (2015) 6:58. 10.3389/fimmu.2015.00058 25729384 PMC4325913

[24] Mondanelli G Iacono A Allegrucci M Puccetti P Grohmann U. Immunoregulatory interplay between arginine and tryptophan metabolism in health and disease. *Front Immunol.* (2019) 10:1565. 10.3389/fimmu.2019.01565 31354721 PMC6629926

[25] Pallotta MT Orabona C Volpi C Vacca C Belladonna ML Bianchi R Indoleamine 2,3-dioxygenase is a signaling protein in long-term tolerance by dendritic cells. *Nat Immunol.* (2011) 12:870–8. 10.1038/ni.2077 21804557

[26] Orabona C Pallotta MT Volpi C Fallarino F Vacca C Bianchi R SOCS3 drives proteasomal degradation of indoleamine 2,3-dioxygenase (IDO) and antagonizes IDO-dependent tolerogenesis. *Proc Natl Acad Sci USA.* (2008) 105:20828–33. 10.1073/pnas.0810278105 19088199 PMC2634889

