# Supplementary material for: Pharmacologic Induction of Endotoxin Tolerance in Dendritic Cells by L-Kynurenine
Source: Front Immunol. 2020 Mar 11;11:292. doi: 10.3389/fimmu.2020.00292 (PMC7081078; doi:10.3389/fimmu.2020.00292)
Supplement: Supplementary file 1 [file Data_Sheet_1.docx]

**SUPPLEMENTARY INFORMATION**

**Pharmacologic induction of endotoxin tolerance in dendritic cells by l-kynurenine**

Giorgia Manni, Giada Mondanelli, Giulia Scalisi, Maria Teresa Pallotta, Dario Nardi, Eleonora Padiglioni, Rita Romani, Vincenzo Nicola Talesa, Paolo Puccetti, Francesca Fallarino* and Marco Gargaro*

***Marco Gargaro**, Ph.D. (marco.gargaro@unipg.it)

***Francesca Fallarino**, Ph.D. (francesca.fallarino@unipg.it)

_____________________________________________________________

University of Perugia

Department of Experimental Medicine

Piazzale Gambuli, n. 1 (C Bldg, 4th Fl)

Perugia 06132, Italy

**This Supplementary File includes:**

Supplementary Figures 1 with Legend

Supplementary Tables 1

**
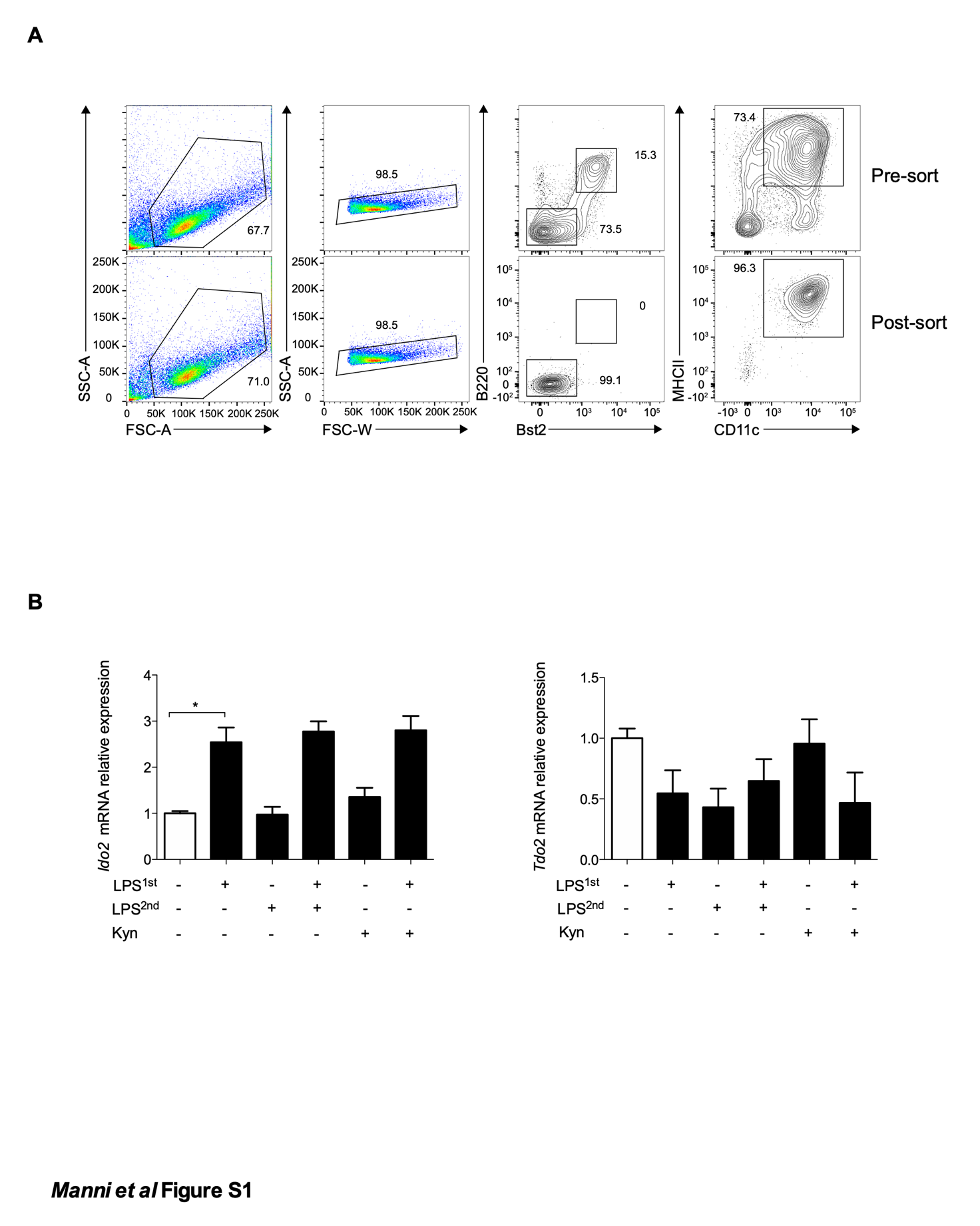
**

**Figure S1 | l-kynurenine does not affect *Ido2* and *Tdo2* expression in LPS-primed-cDCs. A.** Bone marrow cells were analyzed by FACS to determine the relative percentage of pDCs (Bst2^+^ B220^+^) and cDCs (CD11c^+^ MHCII^+^) and the post-sorting purity of the indicated population. Numbers represent the percentage of cells in the indicated gates. **B.** Real-time PCR analysis of *Ido2* and *Tdo2* mRNA expression in WT BMDCs*,* either unprimed or primed with LPS (250 ng/ml) over night (LPS^1st^) and then treated with a second dose of LPS (1 µg/ml) (LPS^2nd^) or l-kynurenine (50 μM) for an additional 24 h. Data (mean ± SD of three experiments) are represented as normalized transcript expression in the samples relative to normalized transcript expression in control cultures. *P < 0.05 (one-way ANOVA).

**Supplementary Table 1. Primer sequences used in this study**

**____________________________________________________________________**

**Gene Primer sequence**

____________________________________________________________________

*β-Actin* F 5’- GGCTCCTAGCACCATGAAGA -3’

R 5’- AGCTCAGTAACAGTCCGCC-3’

*Ido1* F 5’- GTG GGC TTT GCT CTA CCA CA -3’

R 5’- AAG CTG CCC GTT CTC AAT CA -3’

*Ido2* F 5’- GCCCAGAGCTCCGTGCTTCAT -3’

R 5’- TGGGAAGGCGGCATGTAGTCC -3’

*Tdo2* F 5’- GTGAACGACGACTGTCATACCG -3’

R 5’- GCTGGAAAGGGACCTGGAAT -3’

____________________________________________________________________

Abbreviations: F, forward; R, reverse.
